# ω-3 DPA Protected Neurons from Neuroinflammation by Balancing Microglia M1/M2 Polarizations through Inhibiting NF-κB/MAPK p38 Signaling and Activating Neuron-BDNF-PI3K/AKT Pathways

**DOI:** 10.3390/md19110587

**Published:** 2021-10-20

**Authors:** Baiping Liu, Yongping Zhang, Zhiyou Yang, Meijun Liu, Cai Zhang, Yuntao Zhao, Cai Song

**Affiliations:** 1 Research Institute for Marine Drugs and Nutrition, College of Food Science and Technology, Guangdong Ocean University, Zhanjiang 524088, China; liubaiping@stu.gdou.edu.cn (B.L.); zhangyongping@gdou.edu.cn (Y.Z.); zyyang@gdou.edu.cn (Z.Y.); Liumeijun1@stu.gdou.edu.cn (M.L.); zhangcai@gdou.edu.cn (C.Z.); zhaoyt@gdou.edu.cn (Y.Z.); 2 Guangdong Provincial Key Laboratory of Aquatic Product Processing and Safety, College of Food Science and Technology, Guangdong Ocean University, Zhanjiang 524088, China; 3 Marine Medical Research and Development Centre, Shenzhen Institute of Guangdong Ocean University, Shenzhen 518108, China

**Keywords:** omega-3 docosapentaenoic acid, neuroinflammation, neuroprotection, M1/M2 polarizations, microglia-NF-κB/MAPK p38 pathway, BDNF-PI3K/AKT pathway

## Abstract

Microglia M1 phenotype causes HPA axis hyperactivity, neurotransmitter dysfunction, and production of proinflammatory mediators and oxidants, which may contribute to the etiology of depression and neurodegenerative diseases. Eicosapentaenoic acid (EPA) may counteract neuroinflammation by increasing n-3 docosapentaenoic acid (DPA). However, the cellular and molecular mechanisms of DPA, as well as whether it can exert antineuroinflammatory and neuroprotective effects, are unknown. The present study first evaluated DPA’s antineuroinflammatory effects in lipopolysaccharide (LPS)-activated BV2 microglia. The results showed that 50 μM DPA significantly decreased BV2 cell viability after 100 ng/mL LPS stimulation, which was associated with significant downregulation of microglia M1 phenotype markers and proinflammatory cytokines but upregulation of M2 markers and anti-inflammatory cytokine. Then, DPA inhibited the activation of mitogen-activated protein kinase (MAPK) p38 and nuclear factor-κB (NF-κB) p65 pathways, which results were similar to the effects of NF-κB inhibitor, a positive control. Second, BV2 cell supernatant was cultured with differentiated SH-SY5Y neurons. The results showed that the supernatant from LPS-activated BV2 cells significantly decreased SH-SY5Y cell viability and brain-derived neurotrophic factor (BDNF), TrkB, p-AKT, and PI3K expression, which were significantly reversed by DPA pretreatment. Furthermore, DPA neuroprotection was abrogated by BDNF-SiRNA. Therefore, n-3 DPA may protect neurons from neuroinflammation-induced damage by balancing microglia M1 and M2 polarizations, inhibiting microglia-NF-κB and MAPK p38 while activating neuron-BDNF/TrkB-PI3K/AKT pathways.

## 1. Introduction

Long chain omega (n)-3 polyunsaturated fatty acids (PUFAs) have been reported to alleviate depression and neurodegenerative diseases through anti-inflammatory and antioxidant effects [1]. Currently, eicosapentaenoic acid (EPA) and docosahexaenoic acid (DHA) are two of the most studied n-3 PUFAs. However, EPA and DHA exert different therapeutic effects on mood and cognition. For example, clinical studies found that EPA (>1 g/day [2] or higher EPA with a lower dose of DHA [3]) greatly reduced depressive symptoms, but not pure or higher doses of DHA [4]. However, higher doses of DHA with lower doses of EPA could improve age- or mild Alzheimer’s disease (AD)-induced cognitive decline [3,5,6].

In the brain, DHA is the most enriched n-3 PUFA [7], while levels of EPA, which has fast metabolism, are 250–300 times lower than those of DHA [8]. Thus, it is unknown whether the improvement induced by EPA in depression is achieved by itself or via DPA. We previously reported that EPA attenuated Parkinson’s disease-like changes in a 1-methyl-4-phenylpyridinium (MPP+)-induced mouse model by increasing both EPA and n-3 docosapentaenoic acid (DPA) concentrations, but not DHA concentration in the brain. DPA, enriched in the *Clupeidae* family of fish, seals, and salmon, is an intermediate metabolite from EPA forming DHA. A similar result from an in vitro study demonstrated that the anti-inflammatory effects of DPA on activated RAW264.7 cells were independent of DHA conversion [9]. Furthermore, recent evidence suggested that DPA, on one hand, significantly downregulated the mRNA expression of proinflammatory factors, interleukin (IL)-6, IL-1β, tumor necrosis factor (TNF)-α, inducible nitric oxide synthase (iNOS), and cyclooxygenase 2 (COX-2) in RAW264.7 cells stimulated by LPS [9]. On the other hand, DPA increased the expression of anti-inflammatory cytokine IL-10 in a dextran sulphate sodium-induced colitis model [10]. Furthermore, clinical studies reported that DPA concentration in red blood cells was inversely associated with high-sensitivity C-reactive protein and TNF-α, which positively correlated with a lower risk of systemic inflammation in the James Bay Cree population [11,12]. These findings suggested that the antidepressant or neuroprotective effects of EPA were not mediated by DHA production, but rather by EPA or/and DPA. However, most of these studies focused on peripheral inflammation. Whether DPA can counteract neuroinflammation, thereby attenuating depression and neurodegenerative disease, is unknown. Neuroinflammatory response can be induced by activated microglia through nuclear factor-κB (NF-κB) p65 and mitogen-activated protein kinase (MAPK) p38 signaling pathways [13]. Activated microglia can be polarized to a proinflammatory M1 phenotype or an anti-inflammatory M2 phenotype. M1 activation increased proinflammatory mediators and oxidative compounds, including Iba-1, CD68, IL-1β, TNF-α, and nitric oxide (NO) [14], which are important triggers for neuroinflammation. By contrast, M2 phenotype can promote tissue repair through releasing anti-inflammatory mediators such as IL-10 and upregulating M2 markers CD206 and Arg1 expression [15]. However, recent studies have shown that classic M1/M2 polarization was unable to reflect all types of microglial responses in neurodegenerative diseases such as AD or amyotrophic lateral sclerosis. For instance, disease-associated microglia (DAM) appeared in AD models of mice; these microglia cleaned the protease-resistant misfolded and aggregated proteins [16]. Nevertheless, the M1/M2 polarization paradigm is still applicable for in vitro studies even though the results do not directly reflect microglia status in vivo [17]. Therefore, whether and how DPA regulates the balance of M1/M2 polarization and pro-/anti-inflammatory factors through NF-κB p65 and MAPK p38 signaling pathways are unclear.

Neuroinflammation is involved in the pathogenesis of depression and neurodegeneration. With regards to neuroprotective function, a recent study reported that DPA oral administration can attenuate increased sphingomyelinase and caspase 3 activity, oxidative stress, and microglial activation in aged rats, all of which were associated with restoration of long-term potentiation (LTP) and spatial memory [11,18]. Moreover, DPA treatment for 16 days markedly attenuated rat depression-like behavior in a forced swimming test and increased hippocampal DPA levels [19]. Therefore, we supposed that DPA may reduce depressive symptoms and cognitive decline by protecting neurons. However, the cellular and molecular mechanisms by which this would occur are unclear. Brain-derived neurotrophic factor (BDNF) is an important neuroprotective factor that can promote neuronal survival and synaptogenesis and repair brain damage by binding its high affinity receptor TrkB [20]. Moreover, activated BDNF-TrkB stimulates the downstream signaling pathway PI3K/AKT, which is expressed widely in the central nervous system, and promotes neuron cell survival, proliferation and differentiation [21]. However, whether DPA protects neurons by activating BDNF/TrkB-PI3K/AKT pathways is also unknown.

Therefore, the aims of this study were to determine: (1) whether DPA could reverse neuroinflammatory response through anti-inflammatory pathways in a neuroinflammation model, LPS-activated BV2 microglia cells; (2) whether DPA could protect neurons from LPS-activated microglial M1 phenotype through activating BDNF/TrkB mediated PI3K/AKT pathways in the neuron-microglia interacting model.

## 2. Results

### 2.1. LPS-Induced Increases in BV2 Cell Viability and NO Levels Were Ameliorated by DPA

**Figure 1:**
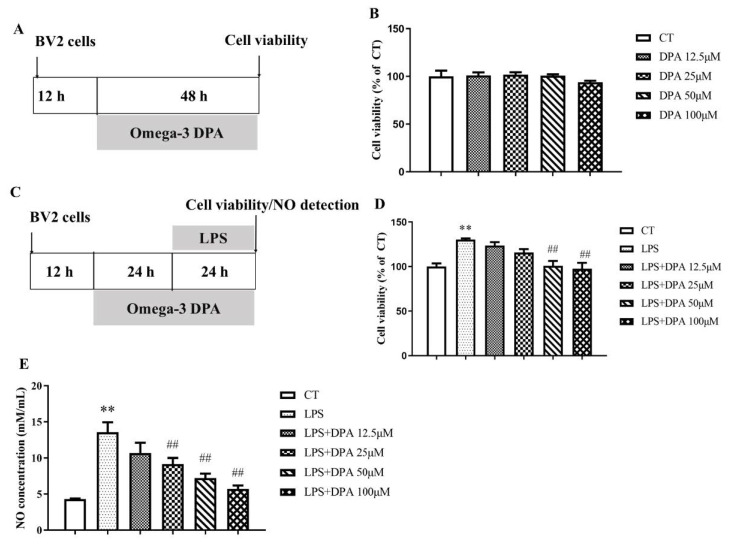
DPA reversed the increase in BV2 cell viability and NO concentration in the BV2 cell supernatant induced by LPS. (**A**,**C**) Experimental procedure. (**B**) Changes in BV2 cell viability after treatment with DPA at different concentrations for 48 h. (**D**) Changes in BV2 cell viability after treatment with 100 ng/mL LPS for 24 h with DPA administration at doses from 12.5 to 100 μM. (**E**) DPA inhibited the increase in NO concentration in the BV2 cell supernatant induced by LPS. The data are expressed as mean ± SEM (n = 6). ** *p* < 0.01 versus CT; ^##^ *p* < 0.01 versus LPS. One-way analysis of variance (ANOVA) followed by Tukey’s post hoc test.

To determine DPA toxicity, BV2 cells were treated with different doses of DPA from 12.5 to 100 μM for 48 h. The results showed that there was no effect of DPA on BV2 cell viability (Figure 1A,B). Administration of LPS at 100 ng/mL for 24 h significantly increased BV2 cell viability (*p* < 0.01) and NO concentration in BV2 cell supernatant (*p* < 0.01). Pretreatment with DPA at 50 μM (all *p* < 0.01) and 100 μM (all *p* < 0.01) significantly reversed the increase in BV2 viability and NO level induced by LPS (Figure 1C–E). Based on the above results, 50 μM DPA was selected for further experiments.

### 2.2. DPA Inhibited LPS-Induced Neuroinflammation by Balancing Microglia M1/M2 Phenotype Polarization

**Figure 2:**
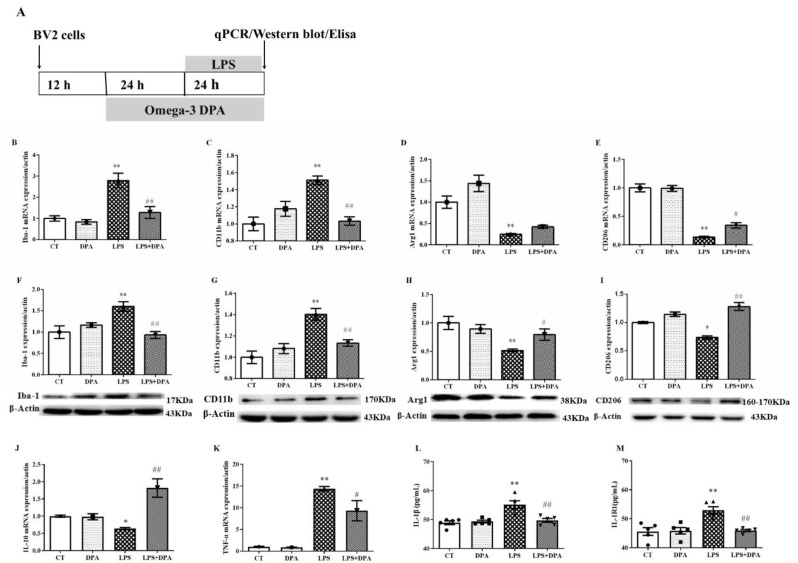
DPA suppressed microglia M1-related proinflammatory response and promoted M2-related anti-inflammatory response: (**A**) Experimental procedure. The mRNA expression of (**B**) Iba-1, (**C**) CD11b, (**D**) Arg1, (**E**) CD206, (**J**) IL-10, and (**K**) TNF-α in LPS-activated BV2 cells. The protein expression of (**F**) Iba-1, (**G**) CD11b, (**H**) Arg1, and (**I**) CD206 in LPS-activated BV2 cells. The concentrations of (**L**) IL-1β and (**M**) IL-1R1 in the supernatant from LPS-activated BV2 cells. The data are expressed as mean ± SEM (n = 5). * *p* < 0.05, ** *p* < 0.01 versus CT; ^#^ *p* < 0.05, ^##^ *p* < 0.01 versus LPS. Triangles, squares and circles represent the expression or concentrations of sample. Two-way ANOVA followed by Tukey’s post hoc test.

At the mRNA level, LPS significantly increased the mRNA expression of microglia M1 phenotype markers Iba-1 (*p* < 0.01) and CD11b (*p* < 0.01) and decreased the expression of M2 markers Arg1(*p* < 0.01) and CD206 (*p* < 0.01). However, pretreatment with 50 μM DPA significantly reversed these changes (Iba-1: *p* < 0.01; CD11b: *p* < 0.01; CD206: *p* < 0.05) except for Arg1 (Figure 2B–E).

At the protein level, pretreatment with 50 μM DPA significantly suppressed the upregulation of the protein expression of microglia M1 markers Iba-1 (*p* < 0.01) and CD11b (*p* < 0.01) while reversing the downregulation of M2 markers Arg1 (*p* < 0.05) and CD206 (*p* < 0.01) in LPS-activated BV2 cells (Figure 2F–I).

With regards to pro- and anti-inflammatory cytokines, LPS significantly increased TNF-α (*p* < 0.01) but decreased IL-10 (*p* < 0.05) mRNA expression. However, DPA significantly reversed these changes (TNF-α: *p* < 0.05; IL-10: *p* < 0.01) (Figure 2J,K).

With regards to other potent proinflammatory cytokines, comparison between groups confirmed that LPS significantly elevated the release of IL-1β (*p* < 0.01) and IL-1R1 (*p* < 0.01). DPA, however, significantly reversed these changes (all *p* < 0.01) (Figure 2L,M).

### 2.3. DPA Inhibited the Activation of NF-κB p65 and MAPK p38 Signaling Pathways in LPS-Activated BV2 Cells

**Figure 3:**
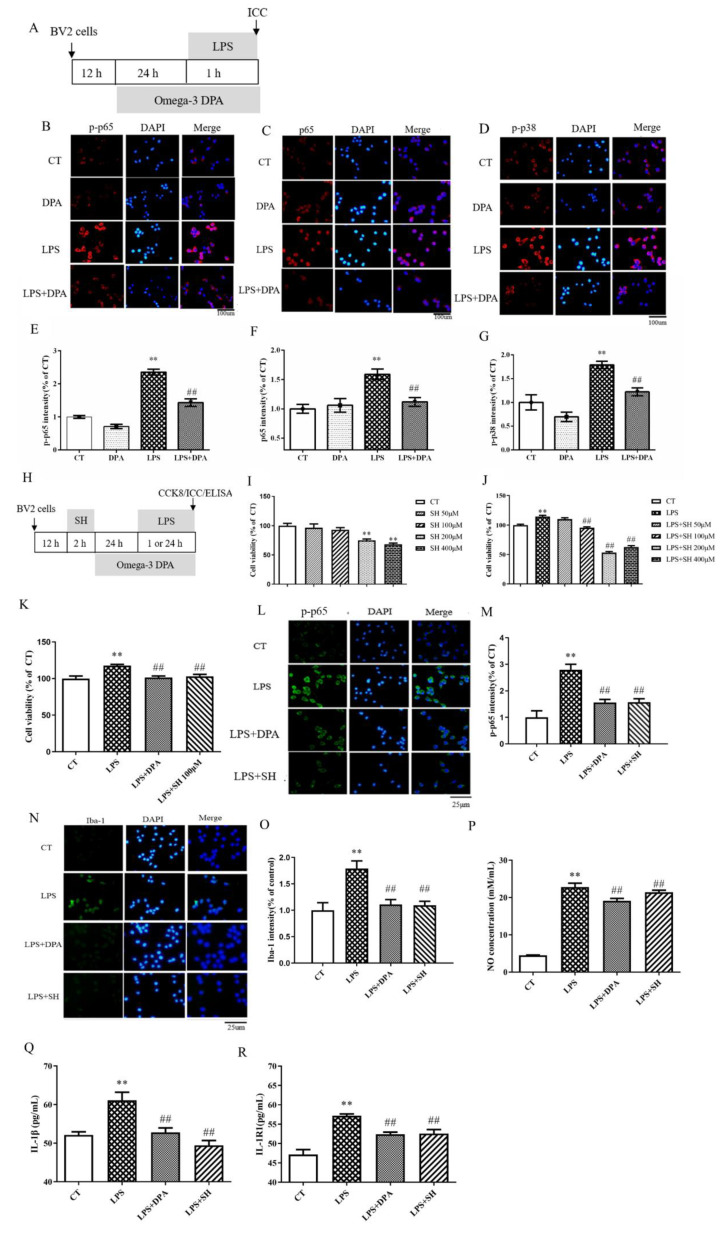
DPA inhibited the activation of NF-κB and MAPK p38 signaling pathways in LPS-activated BV2 cells. (**A**,**H**) Experimental procedure. (**B**–**D**) The representative images immunostained for *p*-p65, p65, and p-p38 (p-p65, p65, and p-p38 (red); DAPI (blue)). (**E**–**G**) p-p65, p65, and p-p38 expression per cell, quantified. (**I**) Changes in BV2 cell viability after treatment with SH at different concentrations for 48 h. (**J**) Changes in BV2 cell viability after treatment with 100 ng/mL LPS for 24 h with or without different concentrations of SH. (**K**) Changes in BV2 cell viability after treatment with 100 ng/mL LPS for 24 h with or without SH or/and DPA administration. (**L**,**N**) The representative images immunostained for p-p65 and Iba-1 (green) and DAPI (blue). (**M**,**O**) p-p65 and Iba-1 expression per cell, quantified. (**P**–**R**) NO, IL-1β, and IL-1R1 concentrations in BV2 cell supernatant. The data are expressed as mean ± SEM (n = 5–6). ** *p* < 0.01 versus CT; ^##^ *p* < 0.01 versus LPS. Results in Figure 3E–G were measured by one-way ANOVA followed by Tukey’s post hoc test; all other results were measured by two-way ANOVA.

LPS significantly increased the expression of *p*-p65 (*p* < 0.01), p65 (*p* < 0.01) and *p*-p38 (*p* < 0.01). This was significantly reversed by DPA pretreatment (all *p* < 0.01) (Figure 3A–G).

To further confirm the effect on the NF-κB p65 signaling pathway, as shown in Figure 3H–J, we used sinomenine hydrochloride (SH), a NF-κB inhibitor, as a positive control reagent. From the results, we found that 100, 200, and 400 μM SH significantly blocked the LPS-induced increase in BV2 cell viability (all *p* < 0.01). However, the administration of 200 and 400 μM SH significantly decreased BV2 cell viability (all *p* < 0.01) when compared to BV2 cells without LPS treatment, which suggested a toxic effect. Therefore, we chose 100 μM SH for the following experiments. The results showed that 100 μM SH could significantly counteract the increase in BV2 cell viability (*p* < 0.01), the expression of *p*-p65 (*p* < 0.01) and Iba-1 (*p* < 0.01), and the concentration of NO (*p* < 0.01), IL-1β (*p* < 0.01), and IL-1R1 (*p* < 0.01) after LPS administration. The effects in the SH group are similar to those found in the DPA group (Figure 3K–R).

### 2.4. DPA Reversed the Downregulation of BDNF/TrkB-PI3K/AKT Signaling Pathway in the Damaged SH-SY5Y Cells Induced by Inflammation

**Figure 4:**
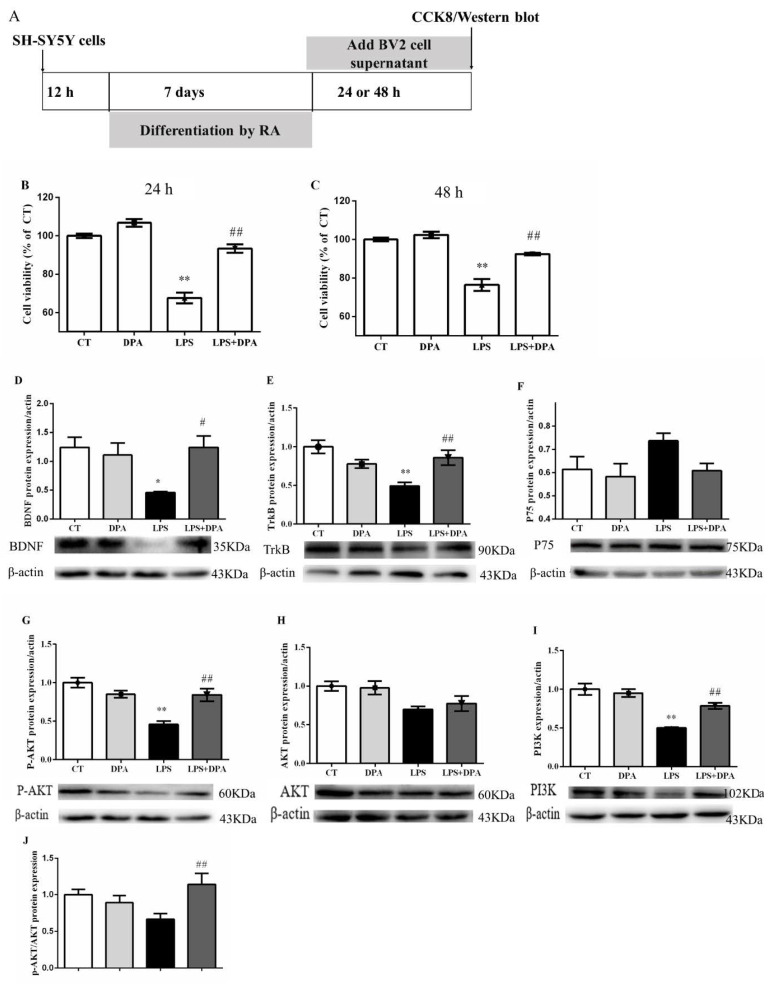
DPA attenuated the downregulation of the BDNF/TrkB-PI3K/AKT signaling pathway in SH-SY5Y cells damaged by conditioned medium from LPS-activated BV2 cells. (**A**) Experimental procedure. DPA attenuated the decrease in SH-SY5Y cell viability induced by supernatant from LPS-activated BV2 cells at 24 h (**B**) and 48 h (**C**). The protein expression of (**D**) BDNF, (**E**) TrkB, (**F**) P75, (**G**) p-AKT, (**H**) AKT, (**I**) PI3K, and (**J**) p-AKT/AKT in SH-SY5Y cells. The data are expressed as mean ± SEM (n = 5). * *p* < 0.05, ** *p* < 0.01 versus CT; ^#^ *p* < 0.05, ^##^ *p* < 0.01 versus LPS. Two-way ANOVA followed by Tukey’s post hoc test.

First, conditioned medium from LPS-activated BV2 cells significantly suppressed SH-SY5Y cell viability at both 24 h (*p* < 0.01) and 48 h (*p* < 0.01). These changes were significantly reversed by the conditioned medium pretreated with DPA (all *p* < 0.01) (Figure 4B,C).

Second, supernatant from LPS-activated BV2 cells significantly decreased the protein expression of BDNF (*p* < 0.05) and TrkB (*p* < 0.01) in SH-SY5Y cells compared to the control group. These changes were significantly reversed by the conditioned medium pretreated with DPA (BDNF: *p* < 0.05; TrkB: *p* < 0.01) (Figure 4D–F).

Lastly, the conditioned medium from LPS-stimulated BV2 cells significantly decreased the protein expression of *p*-AKT (*p* < 0.01) and PI3K (*p* < 0.01) in SH-SY5Y cells compared to the control group. These changes were significantly reversed by the conditioned medium pretreated with DPA (all *P* < 0.01) (Figure 4G–I).

### 2.5. BDNF SiRNA Blocked the Effect of DPA on the Dysfunction of BDNF System and PI3K/AKT Signaling Pathway in the Damaged SH-SY5Y Cells Induced by Inflammation

**Figure 5:**
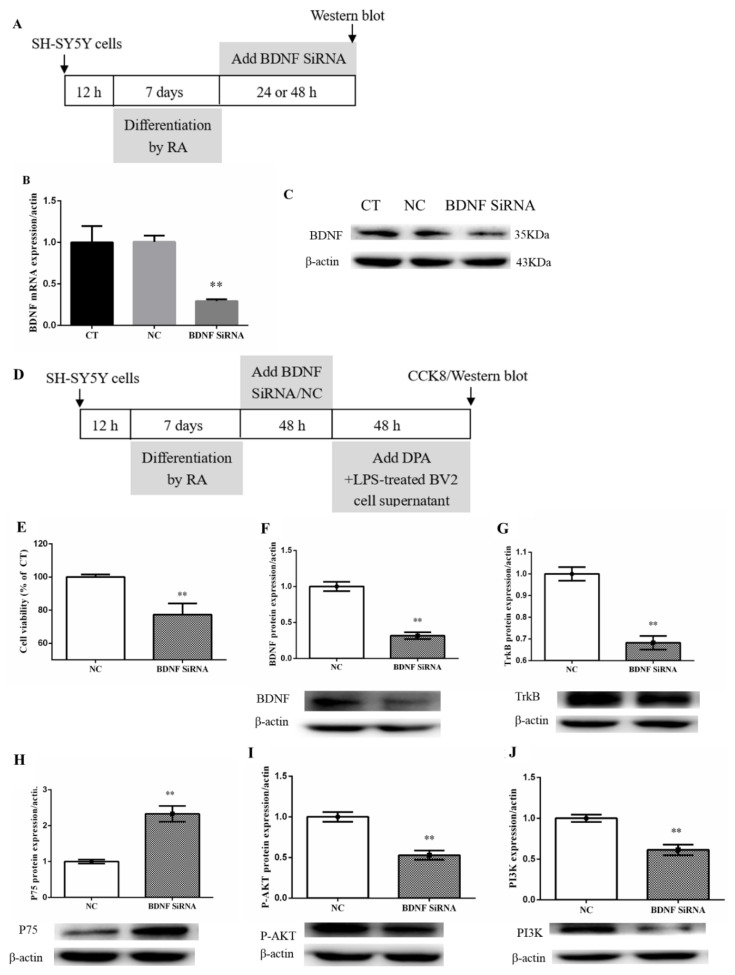
BDNF SiRNA blocked DPA to restore the dysfunction of the BDNF system and PI3K/AKT signaling pathway in SH-SY5Y cells induced by conditioned medium from LPS+DPA-activated BV2 cells. (**A**,**D**) Experimental procedure. BDNF SiRNA suppressed the (**B**) mRNA expression and (**C**) protein expression of BDNF. (**E**) BDNF SiRNA decreased SH-SY5Y cell viability after treatment with conditioned medium from LPS+DPA-stimulated BV2 cells for 48 h. (**C**) Protein expression of (**F**) BDNF, (**G**) TrkB, (**H**) P75, (**I**) p-AKT, and (**J**) PI3K in SH-SY5Y cells. The data are expressed as mean ± SEM (n = 3–5). ** *p* < 0.01 versus NC. The results were analyzed by Student’s *t*-test.

After BDNF SiRNA treatment, the mRNA (*p* < 0.01) and protein expression of BDNF significantly decreased in SH-SY5Y cells (Figure 5A–C).

After BDNF SiRNA or NC treatment for 48 h, supernatant from DPA+LPS-treated BV2 was added to SH-SY5Y cell culture wells for following experiments. The results showed that BDNF SiRNA treatment blocked DPA reversed the decrease in cell viability (*p* < 0.01) and the protein expression of BDNF (*p* < 0.01), TrkB (*p* < 0.01), p-AKT (*p* < 0.01) and PI3K (*p* < 0.01) in SH-SY5Y cells when compared to the NC group; and significantly increased P75 (*p* < 0.01) expression (Figure 5D–J).

## 3. Discussion

As mentioned in the introduction, previous studies have reported that n-3 PUFAs, mainly EPA and DHA, could improve mood and cognition by protecting neurons from inflammation and oxidative stress-induced apoptosis [22,23,24]. However, the effect of DPA, an intermediate metabolite between EPA and DHA, on affective or neurodegenerative diseases is almost unknown. The current in vitro study, for the first time, demonstrated that DPA could significantly exert antineuroinflammatory and neuroprotective effects, which may provide a potential treatment for depression, temporal lobe epilepsy [25], and neurodegenerative diseases. Several new findings are discussed below.

### 3.1. n-3 DPA Shifted Microglia from M1 to M2 Polarization and Inhibited the Activation of NF-κB and MAPK p38 Signaling Pathways

The contribution of neuroinflammation to neurological disorders has been extensively demonstrated [26,27,28]. As a trigger of neuroinflammation, activated microglia result in imbalance between the release of proinflammatory and anti-inflammatory mediators; this balance depends on microglia polarization into M1 or M2 phenotype. The M1 phenotype is characterized by increasing the expression of proinflammatory cytokines and M1 markers, including Iba-1, CD11b, CD68, IL-1β, IL-6, and TNF-α [29,30]. On the contrary, the M2 phenotype can produce anti-inflammatory cytokines and M2 markers such as Arg-1, CD206, IL-4, and IL-10 [31,32]. We and others have previously reported that n-3 PUFAs, especially EPA, exerted antineuroinflammatory properties in vivo in animal models of Alzheimer’s disease, Parkinson’s disease, and IL-1β induced neuroinflammation [33,34]. One mechanism by which this occurred was balancing M1 and M2 polarizations through sirtuin1-mediated deacetylation of the high mobile group box 1 (HMGB1)/NF-κB pathway, leading to a neuroprotective effect [35]. The present study showed that LPS significantly increased BV2 cell viability, which was associated with increased expression of the inflammatory M1 phenotype markers Iba-1 and CD11b; increased concentrations or expression of the proinflammatory cytokines IL-1β, TNF-α, and IL-1R1; and increased oxidant NO production, but also with decreased expression of the anti-inflammatory M2 phenotype markers Arg1 and CD206 and the anti-inflammatory cytokine IL-10. Pretreatment with DPA significantly reversed the aforementioned changes, which results are similar to those of EPA pretreatment.

The present study further revealed that the molecular mechanism involved in DPA anti-inflammatory procedure worked through the modulation of the NF-κB and MAPK p38 signaling pathways. The study showed that LPS significantly increased the expression of p-p65 and p65, indicating a significant activation of the NF-κB signaling pathway. It was reported that activated NF-κB pathway can promote proinflammatory mediator production and NO synthase transcription [36] by binding the promoter region of κB sequences in the nucleus. Thus, DPA reversed the increase in p-p65 and p65 expression induced by the inflammation response, suggesting an inhibitory effect of DPA on NF-κB activity. Furthermore, this finding was demonstrated by administration of positive control reagent SH, a NF-kB inhibitor, to block the NF-κB pathway. The results showed that 100 μM SH could significantly counteract increases in BV2 cell viability, p-p65 and Iba-1 expression, and concentration of NO, IL-1β, and IL-1R1 after LPS administration. These results indicate that the effects of SH were similar to those of DPA, which may suggest that DPA’s anti-inflammatory effect worked by inhibiting the NF-κB pathway.

In addition, MAPKs can also respond to stress stimuli and regulate signaling networks and the biosynthesis of proinflammatory cytokines such as TNF-α and IL-1β [37,38,39]. The activation of the MAPK p38 pathway was observed in the postmortem brains of patients with neurodegenerative diseases and animal models [40]. Recently, MAPK p38 inhibitors have been claimed as novel and potential therapeutics for neurodegenerative diseases [41]. The present study demonstrated that LPS significantly increased p-p38 expression, and that DPA exerted an inhibitory effect in MAPK p38 activity. Therefore, the antineuroinflammatory effect of DPA may be achieved by blocking both the NF-κB and MAPK p38 signaling pathways.

### 3.2. n-3 DPA Protected Neurons by Upregulating BDNF-TrkB Expression

Promotion of microglial M2 and astrocyte A2 phenotypes can directly enhance neurotrophin synthesis and the expression and function of neurotrophin receptors, which protect neuronal survival or prevent neuronal apoptosis [42]. Deficient neurotrophic factors have been directly correlated to neuropathology and symptoms of neurodegenerative diseases or depression. A study in an ageing rat model found that the administration of 120 ng/day BDNF into the medial entorhinal cortex significantly improved spatial learning and memory in water maze performance [43]. Another study showed that BDNF microinjection into the hippocampus significantly decreased neuroinflammation in a type 1 diabetes mouse model [44]. Our previous studies demonstrated that EPA could normalize BDNF-TrkB, NF-κB, and apoptosis signaling pathways [45]. The present in vitro study showed that conditioned medium from LPS-activated BV2 cells significantly decreased SH-SY5Y cell viability and protein expression of BDNF and TrkB, which effects were attenuated by conditioned medium from BV2 cells pretreated with DPA. These results suggested that DPA protected neurons by restoring the dysfunction of the BDNF-TrkB system in damaged SH-SY5Y cells.

### 3.3. n-3 DPA Exerted Neuroprotective Effect by Activating BDNF/TrkB-Mediated PI3K/AKT Pathway

The current study found that supernatant pretreated with DPA largely reversed the downregulated protein expression of p-AKT and PI3K induced by supernatant from LPS-stimulated BV2 cells. It is known that activated Trk receptors can trigger PI3K and subsequently activate AKT [46] by engaging Shc associated with Grb2 and Gab1. The PI3K/AKT signaling pathway not only mediates cell survival [47,48] but benefits axonal growth in sensory neurons via phosphorylating and deactivating GSK-3β activity [46,49,50]. Moreover, these results were further confirmed by the administration of BDNF SiRNA, which reversed the increase in BDNF, TrkB, P-AKT, and PI3K protein expression and the decrease in P75 protein expression after DPA administration. Thus, the data demonstrated that the neuroprotective mechanism of DPA may be achieved by targeting the BDNF/TrkB-mediating PI3K/AKT signaling pathway.

### 3.4. Possible Differences among EPA, DPA and DHA

EPA forms DHA by producing an intermediate metabolite, DPA, which is partially retroconverted to EPA in the liver and brain. However, the endogenous synthesis of EPA and DPA is lower than that of DHA in the brain [51,52]. Recently, increasing evidence has shown unique or different functions of EPA, DPA, and DHA. As mentioned above, EPA (0.8% in diet) markedly attenuated the symptom of Parkinson’s disease induced by MPP^+^, which was associated with significantly increased levels of EPA and DPA, but not DHA, in the brain [53]. Furthermore, an important study compared the effects of EPA and DPA on microglial activity, oxidative stress, cognitive function, and synaptic plasticity in aged rats. The results showed that EPA and DPA equally protected neurons in terms of the aforementioned parameters [14]. However, DPA induced a stronger increase in anti-inflammatory cytokine IL-10 levels than EPA and DHA in a colitis model induced by dextran sulphate sodium [10]. Although the present study demonstrated antineuroinflammatory and neuroprotective effects of DPA, the differences between DPA and EPA or DHA, as well as whether the neuroprotective effect of EPA works by itself or via DPA, need to be further explored.

Taken together, this study, for the first time, demonstrated that (1) DPA shifted microglia from the proinflammatory M1 into anti-inflammatory M2 phenotype, reflected by decreased proinflammatory cytokines and oxidants, such as TNF-α, IL-1β, and NO, and increased anti-inflammatory cytokine IL-10. The mechanism was demonstrated through inhibiting NF-κB and MAPK p38 signaling pathways; (2) DPA protected neuronal cells against neuroinflammation induced by conditioned medium from LPS-stimulated BV2 cells. The underlying mechanisms were related to the upregulation of neuron-BDNF/TrkB-PI3K/AKT signaling pathways. Therefore, DPA may be considered as a potential treatment for depression or neurological diseases.

## 4. Materials and Methods

### 4.1. Experimental Design

**Figure 6:**
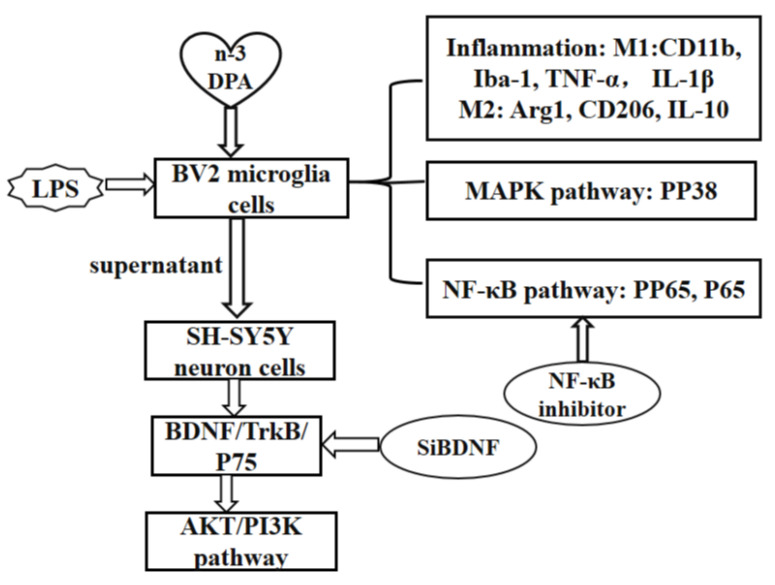
Experimental design.

Please find the specific experiment design in Figure 6.

### 4.2. Cell Culture and Differentiation

BV2 microglia cells were obtained from the Institute of Biochemistry and Cell Biology (Shanghai, China) and cultured in DMEM/high medium (Hyclone, Logan, UT, USA) supplemented with 10% fetal bovine serum (FBS, Gibco, Grand Island, NY, USA) and 1% penicillin-streptomycin (Gibco, USA) at 37 °C in a 5% CO_2_/95% air incubator [54].

SH-SY5Y cells were obtained from the same place and cultured in MEM/F12 medium (MEM medium: F12 medium 1:1, Gibco, USA) supplemented with 15% fetal bovine serum (FBS, Gibco, USA) and 1% penicillin–streptomycin (Gibco, USA) at 37 °C in a 5% CO_2_/95% air incubator. SH-SY5Y cells were differentiated into fully human neuron-like cells by treatment with all-trans-retinoic acid (RA, Sigma, Louis, MO, USA) at a final concentration of 10 μM for 7 days (medium was changed every two days) [23].

### 4.3. Neuron–Microglia Cell Interaction

The supernatant was collected after BV2 cells were stimulated by 100 ng/mL LPS and/or DPA, and then differentiated SH-SY5Y cells were cultured with the supernatant for following measurements.

### 4.4. BDNF SiRNA Preparation and Transfection

Human BDNF-specific SiRNA and negative control SiRNA were purchased from GenePharma (Shanghai, China). SH-SY5Y cells were seeded at a density of 1 × 10^5^ cells/mL and transfected with BDNF-specific SiRNA or negative control SiRNA using transfection reagents (GenePharma, Shanghai, China) according to the manufacturer’s instructions after SH-SY5Y cells were differentiated with RA. After 24 or 48 h of transfection, the efficiency of SiRNA was detected by q-PCR and Western blot analysis.

### 4.5. Cell Viability Assay

After BV2 cells were treated by LPS and/or DPA, or SH-SY5Y cells were treated by LPS and/or DPA-activated BV2 cell supernatant, for 24 or 48 h, the cell counting kit (CCK-8, APEXBIO, Houston, TX, USA) reagent (10 μL) was added into each well, diluted by 100 μL culture medium, and incubated for 3 h according to the manufacturer’s instructions. The absorbance was measured at 450 nm with a 96-well plate reader.

### 4.6. Measurement of Nitric Oxide

BV2 cells were seeded in the plates for 12 h, pretreated with different concentrations of DPA (0, 12.5, 25, 50, or 100 μM) for 24 h, and incubated with 100 ng/mL LPS for 24 h. Fifty microliters of cell supernatant was added with 50 μL sulfanilamide solution and incubated 10 min at room temperature in dark conditions. Then, 50 μL NED solution was added to all wells and incubated at room temperature for 10 min, again in dark conditions. Absorbance was measured within 30 min in a plate reader at 570 nm. A standard curve was generated in the same manner as for NO_2_^−^ quantitation. The NO concentration in the cell supernatant was calculated according to the standard curve.

### 4.7. Measurement of mRNA Expression with Quantitative PCR

The total mRNA was extracted from cells using the Trizol reagents following a manufacturer’s protocol. cDNA was synthesized from 2 μg total RNA using an RT kit (Promega, Madison, Wisconsin, USA). PCR amplifications were performed on a CFX96Touch^TM^ Real-Time PCR Detection System using SYBR^®^ Green Master Mix kit at conditions of initial activation at 95 °C for 30 s, followed by 40 cycles of amplification (95 °C for 5 s, 60 °C for 30 s) [55]. The primer sequences for target genes and the internal control, β-actin, are listed in Table 1. Fold change was calculated using the 2^−ΔΔCt^ method. The values were normalized against the endogenous control, β-actin.

### 4.8. Measurement of Protein Expression by Western Blot

The total protein was extracted from harvested cells, and the concentration was measured with the BCA protein quantitative kit (Dingguo, Beijing) according to the manufacturer’s protocol. Thirty micrograms of total proteins were separated by 10% SDS-PAGE gels and transferred onto PVDF membranes (Millipore, Bedford, MA, USA). After blocking with 5% nonfat milk for 2 h at room temperature, membranes were incubated with primary and secondary (1:5000, MultiSciences, Hangzhou, China) antibodies [30]. The primary antibodies included Iba-1 (1:200, sc-32725, Santa Cruz, CA, USA), CD11b (1:200, sc-6612, Santa Cruz, USA), Arg1 (1:200, sc-271430, Santa Cruz, USA), BDNF (1:1000, Sangon, Shanghai, China), TrkB (1:1000, #46035, CST, Boston, Massachusetts, USA), P75 (1:400, sc-271708, Santa Cruz, USA), P-AKT (1:1000, #40605, CST, USA), AKT (1:1000, #46855, CST, USA), PI3K (1:200, sc-365404, Santa Cruz, USA), and β-Actin (1:400, sc-8432, Santa Cruz, USA). The proteins on the membranes were detected by enhanced chemiluminescence (ECL) (Millipore Corp., USA). The bands were analyzed by a chemiluminescence system (Tanon 5200, Shanghai, China). All target proteins were quantified by normalizing them to β-Actin reprobed on the same membrane and calculated as a percentage of the control group.

### 4.9. Immunofluorescence

Following the culture and treatments, cells were fixed with 4% paraformaldehyde (Solarbio, Beijing, China) and blocked with 5% normal goat serum (MultiSciences, Hangzhou, China) in 0.3% Triton-X-PBS and then immunostained for p65 (1:100, sc-71675, Santa Cruz, USA), p-p65 (1:100, sc-166748, Santa Cruz, USA), p-p38 (1:100, sc-166182, Santa Cruz, USA), and Iba-1 (1:100, sc-32725, Santa Cruz, USA). Alexa Fluor 594-conjugated goat antimouse IgG (1:200, ab150116, Abcam, Cambridge, England) was used as secondary antibody. DAPI (Biomol, Hamburg, Germany) was used for counterstaining [54]. Fluorescence images were captured by a fluorescence microscope system (OLYMPUS IX73, Olympus, Japan). The images were analyzed with software Image J (Image J Version 1.52e, National Institutes of Health, Bethesda, MD, USA).

### 4.10. Measurement of IL-1β and IL-1R1 Concentrations

BV2 cells were seeded in plates for 12 h followed by pretreatment with 50 μM DPA for 24 h. After incubation with 100 ng/mL LPS for 24 h, cell supernatants were harvested. The concentrations of IL-1β and IL-1R1 were measured by ELISA with commercial reagent kits purchased from Beijing Dongge Biotechnology Co. Ltd (Beijing, China) following the manufacturer’s instructions.

### 4.11. Statistical Analysis

Results are expressed as mean ± standard error of mean (SEM). Statistical analysis was performed using Graphpad Prism 6 software. BV2 cell viability, NO concentration, and data from the NF-κB inhibitor experiment were analyzed by one-way ANOVA. The results from the BDNF SiRNA experiment were analyzed by Student’s t-test. The rest of data were analyzed by two-way ANOVA (two factors: model and treatment). Differences between groups were assessed by Tukey’s post hoc test. Significance was set at *p* < 0.05.

## Figures and Tables

**Table 1:** Primer sequences of quantitative PCR.

Gene Name	Primer Sequences (5′–3′)
CD11b	F: CCCATGACCTTCCAAGAGAA
	R: AGAGGGCACCTGTCTGGTTA
Iba-1	F: AAGTCAGCCAGTCCTCCTCAGC
	R: CCAGGCATCACTTCCACATCAGC
CD68	F: CCTCTTGCTGCCTCTCATCATTGG
	R: GGCTGGTAGGTTGATTGTCGTCTG
Arg1	F: TAACCTTGGCTTGCTTCGGAACTC
	R: TGGCGCATTCACAGTCACTTAGG
CD206	F: ACCTGGCAAGTATCCACAGCATTG
	R: TGTTGTTCTCATGGCTTGGCTCTC
IL-10	F: CAAGGCAGTGGAGCAGGTGAAG
	R: GCTCTGTCTAGGTCCTGGAGTCC
TNF-α	F: GCGACGTGGAACTGGCAGAAG
	R: GCCACAAGCAGGAATGAGAAGAGG
BDNF	F: GGAGACACATCCAGCAAT
	R: ACAAGAACGAACACAACAG
β-actin	F: CATCCGTAAAGACCTCTATGCCAAC R: ATGGAGCCACCGATCCACA

## Data Availability

All data are contained within this article.
